# Traumatic Spinal Cord Infarction Associated With Elevated Intracranial and Intraspinal Pressure in a Pediatric Patient: A Case Report and Review of the Literature

**DOI:** 10.7759/cureus.101846

**Published:** 2026-01-19

**Authors:** Julianna L Barbaro, Priyanka Shah, Adam Donithan, Anthony Staples, Mandeep Rana

**Affiliations:** 1 Medicine, Virginia Tech Carilion School of Medicine, Roanoke, USA; 2 Neurology, Edward Via College of Osteopathic Medicine, Blacksburg, USA; 3 Radiology, Virginia Tech Carilion School of Medicine, Roanoke, USA; 4 Pediatrics, Virginia Tech Carilion School of Medicine, Roanoke, USA

**Keywords:** anterior spinal artery, opening pressure, owl's eye sign, pediatric, spinal cord ischemia

## Abstract

Spinal cord ischemia (SCI) in the pediatric population is rare and not well characterized. Initial diagnosis is often delayed, and there are no standardized, evidence-based management strategies. Risk factors and etiologies vary and include trauma, thrombotic or embolic disease, infection, vasculitis, cerebellar herniation, arteriovenous malformation, cardiovascular interventions, scoliosis correction, sickle cell disease, and idiopathic causes. We present a case of an 11-year-old girl with anterior spinal artery (ASA) syndrome who presented four days after a fall with back pain, bilateral lower extremity weakness, and numbness. On examination, she demonstrated objective weakness and decreased pain and temperature sensation (worse on the left than the right) in her lower extremities. Diagnostic workup revealed a thoracic ASA infarction, thoracic transverse process fracture, lumbar paraspinal muscle strain, and elevated intracranial and intraspinal pressure potentially resulting from the recent fall. Notably, the patient’s opening pressure during a fluoroscopy-guided lumbar puncture was markedly elevated, a finding not previously documented in the literature or followed clinically. Owing to the rarity of SCI and its imaging similarities with early-phase myelitis, considerable workup is required for patients presenting with objective weakness to ensure accurate diagnosis and prevent adverse outcomes. Unfortunately, accurate diagnosis of SCI is frequently delayed until irreversible damage has occurred. The ASA infarct and paraspinal muscle strain presented here were diagnosed by MRI.

## Introduction

Spinal cord ischemia (SCI) is a rare but serious condition that can cause long-lasting deficits requiring multidisciplinary care. In adults, it accounts for approximately 1% of all strokes, whereas in children, SCI is so uncommon that its incidence is unknown [1]. SCI is responsible for roughly 6% of acute myelopathies and 1-2% of vascular neurological pathologies [2].

Currently, few reports exist in the pediatric literature regarding SCI diagnosis, etiology, prognosis, and treatment. Pediatric SCI risk factors include blood flow obstruction associated with cardiovascular malformations, cerebellar herniation, iatrogenic or traumatic vascular injury, infection, thrombosis or embolus, and vasculitis [3]. The presentation of SCI can be highly variable, ranging from minor weakness to tetraplegia, which makes accurate diagnosis challenging [4].

We present a pediatric case of anterior spinal artery (ASA) syndrome likely secondary to a fall. This case uniquely contributes to the literature because the patient demonstrated markedly elevated opening pressure (OP) on lumbar puncture (LP) despite the absence of inflammatory markers in CSF, traumatic brain injury, hydrocephalus, or mass lesion, an observation not previously described.

## Case presentation

An 11-year-old girl with a medical history of asthma, attention deficit hyperactivity disorder, and prediabetes presented to an outside ED with sudden-onset bilateral lower extremity (BLE) numbness, weakness, and pain.

The patient reported falling from approximately six feet, landing on her feet, and then falling backward, hitting her head. At the time of injury, she denied loss of consciousness or back pain. Three days after the fall, her ambulation progressively worsened until she could no longer walk, prompting her initial ED visit. After a negative CT scan and X-ray, she was discharged but presented the following day to our ED and was subsequently admitted.

Neurological assessment revealed normal mental status, no papilledema, and intact strength and deep tendon reflexes in the bilateral upper extremities (BUE). Motor strength in the right lower extremity (RLE) ranged from 2/5 (hip flexion) to 4/5 (ankle mobility), whereas the left lower extremity (LLE) was predominantly 0/5. Reflexes were 2+ in the BLE patellar and LLE Achilles, but 1+ in the RLE Achilles, with a positive Babinski sign bilaterally. Sensory examination was intact to light touch and vibration in all four extremities. Pain and temperature sensation were normal in the BUE but decreased in the BLE, with complete loss of sharp and temperature sensation below the thoracic vertebrae T11-T12. Finger-to-nose testing was intact with no tremor.

The patient remained paraplegic on admission and for the first four days of hospitalization. The neurological differential diagnosis included acute myelitis, acute demyelinating process, and SCI.

**Figure 1:**
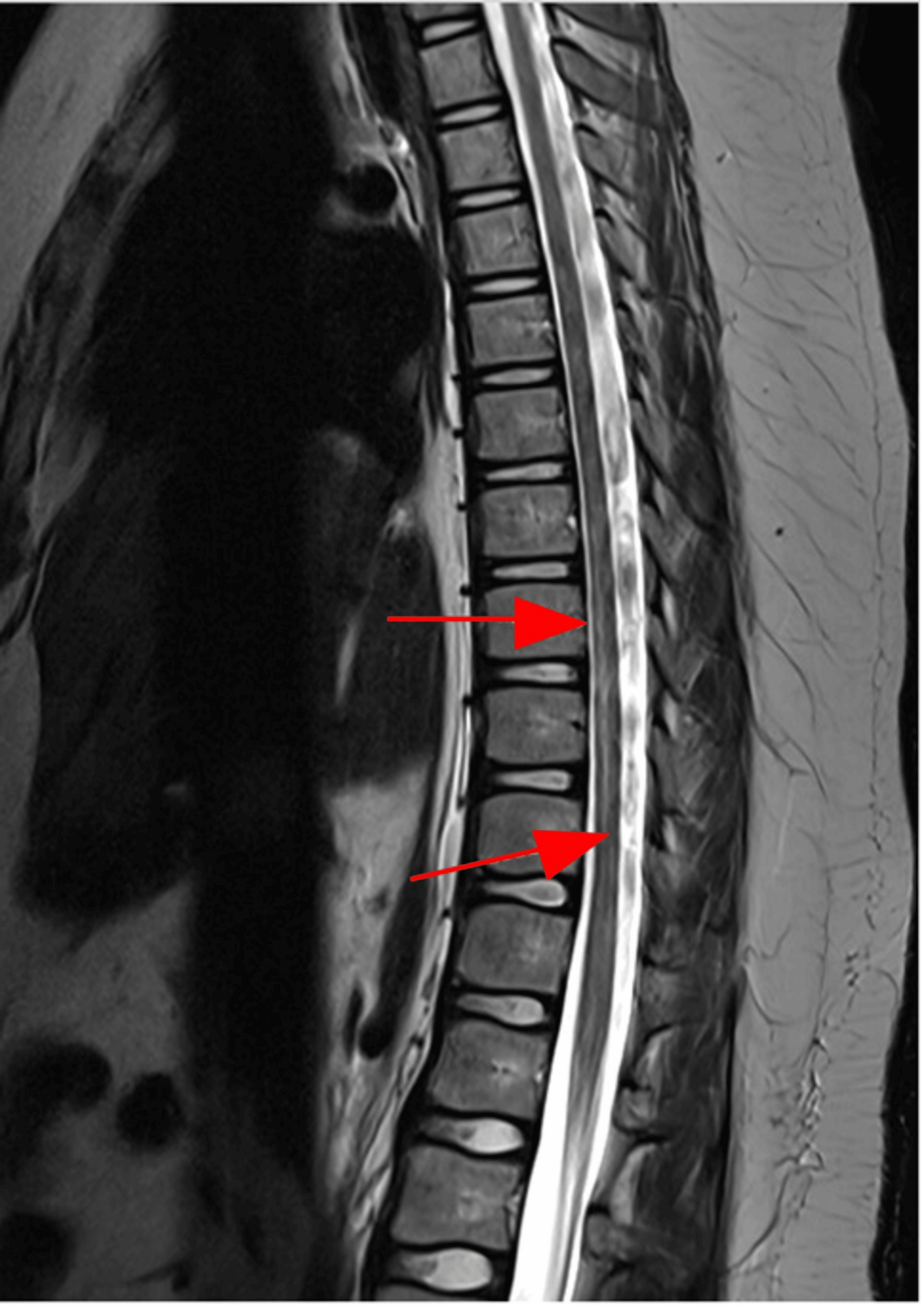
Sagittal T2-weighted MRI of the thoracic spine showing ventral-dominant cord signal abnormality from T8 to T9 through T11 to T12, indicated by red arrows

**Figure 2:**
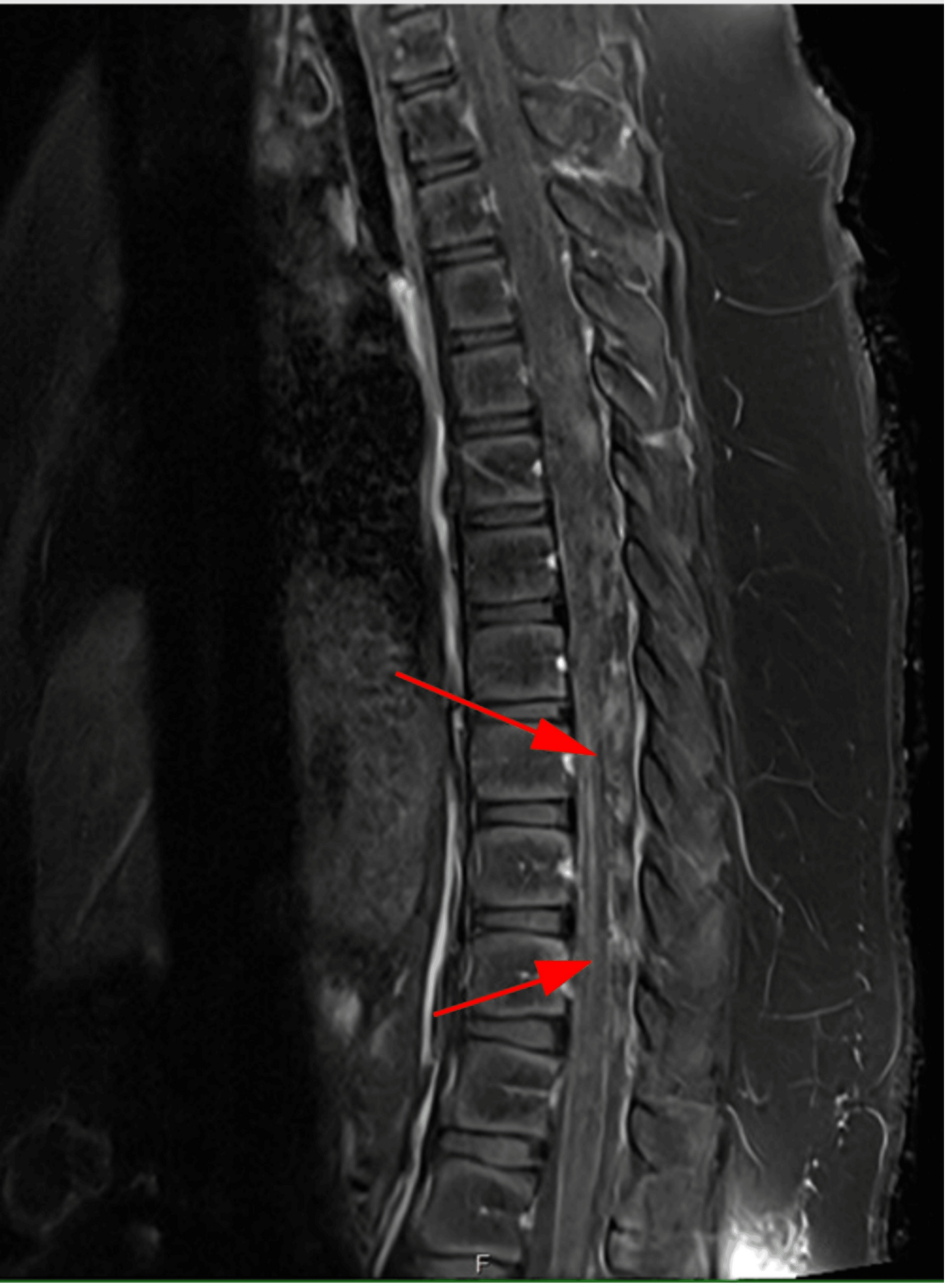
Sagittal post-contrast T1-weighted MRI of the thoracic spine showing no evidence of enhancement from T8 to T9 through T11 to T12, indicated by red arrows

MRI of the brain and cervical (C) spine revealed no abnormalities. MRI of the thoracic (T) spine demonstrated symmetric, ventral-predominant cord signal abnormality from T8 to T12 (Figure 1, Figure 2).

**Figure 3:**
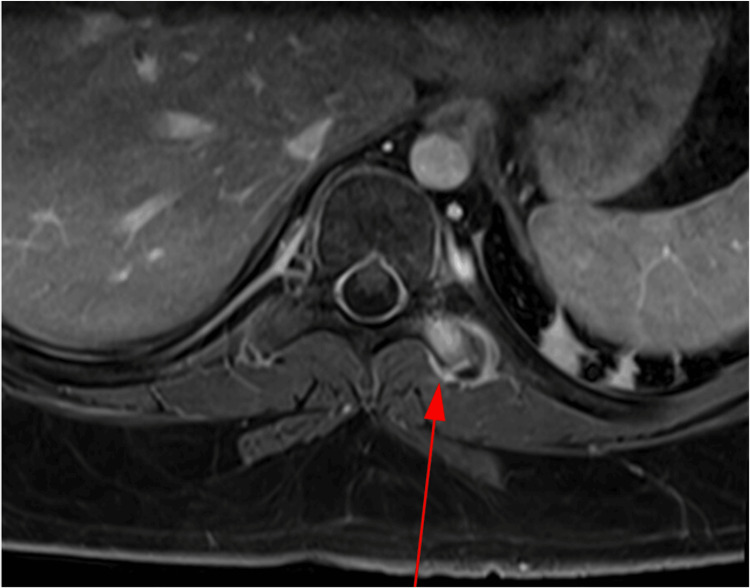
Axial T1-weighted MRI of the thoracic spine The red arrow demonstrates a left transverse process fracture, corroborated on CT of the thoracic spine (not shown).

Imaging of the thoracic spine revealed marrow edema and enhancement of the left T9-T10 transverse process and surrounding tissue, consistent with an acute fracture (Figure 3). MRI of the lumbar (L) spine demonstrated a left-sided L2-L4 paraspinal muscle strain. The radiological differential diagnosis included SCI versus transverse myelitis.

Cardiac evaluation and coagulation profile were normal, and other laboratory investigations were non-contributory (Table 1).

**Table 1:** Additional test results ANA, antinuclear antibody; HbA1c, glycated hemoglobin; HDL, high-density lipoprotein; LDL, low-density lipoprotein; MOG, myelin oligodendrocyte glycoprotein antibody; TSH, thyroid-stimulating hormone

Variables	On admission	Reference range	Unit
White blood cells	7.6	4.5-13.5	K/µL
Hemoglobin	12.2	11.5-15.5	g/dL
Platelets	301	130-400	K/µL
Factor V (Leiden) mutation	Negative	Negative	N/A
Factor VIII	91	60-200	%
Fibrinogen	324	175-425	mg/dL
Protein S activity	112	60-140	%
D-dimer	0.39	<0.5	µg FEU/mL
Antithrombin III	116	80-135	%
Total protein	7.3	6.4-8.3	g/dL
Albumin	4.5	3.2-5.0	g/dL
Aspartate aminotransferase	22	15-37	U/L
Alanine aminotransferase	27	10-49	U/L
HbA1c	5.7	<5.7	%
Cholesterol	143	<170	mg/dL
LDL	91	<110	mg/dL
HDL	34	>45	mg/dL
Glucose	84	60-99	mg/dL
CRP	<0.50	<1.0	mg/dL
TSH	0.572	0.64-6.27	µIU/mL
ANA	Negative	Negative	N/A
Serum MOG antibody	Negative	Negative	N/A
Serum aquaporin-4 antibody	Negative	Negative	N/A
CSF oligoclonal bands	Absent	Absent	N/A
CSF autoimmune encephalopathy panel	Negative	Negative	N/A
CSF meningoencephalitis panel	Negative	Negative	N/A

Neurosurgery evaluated the patient, and, in the absence of compressive pathology or spinal instability, no surgical intervention was indicated. The patient subsequently underwent a fluoroscopy-guided LP, with opening and closing pressures of 54 and 32 cm H₂O, respectively. CSF analysis was normal, without evidence of inflammation (Table 1), and fundoscopy revealed no papilledema despite the markedly elevated OP. Given concerns for transverse myelitis, she was treated with IV methylprednisolone (MPS) 1,000 mg daily for five days.

**Figure 4:**
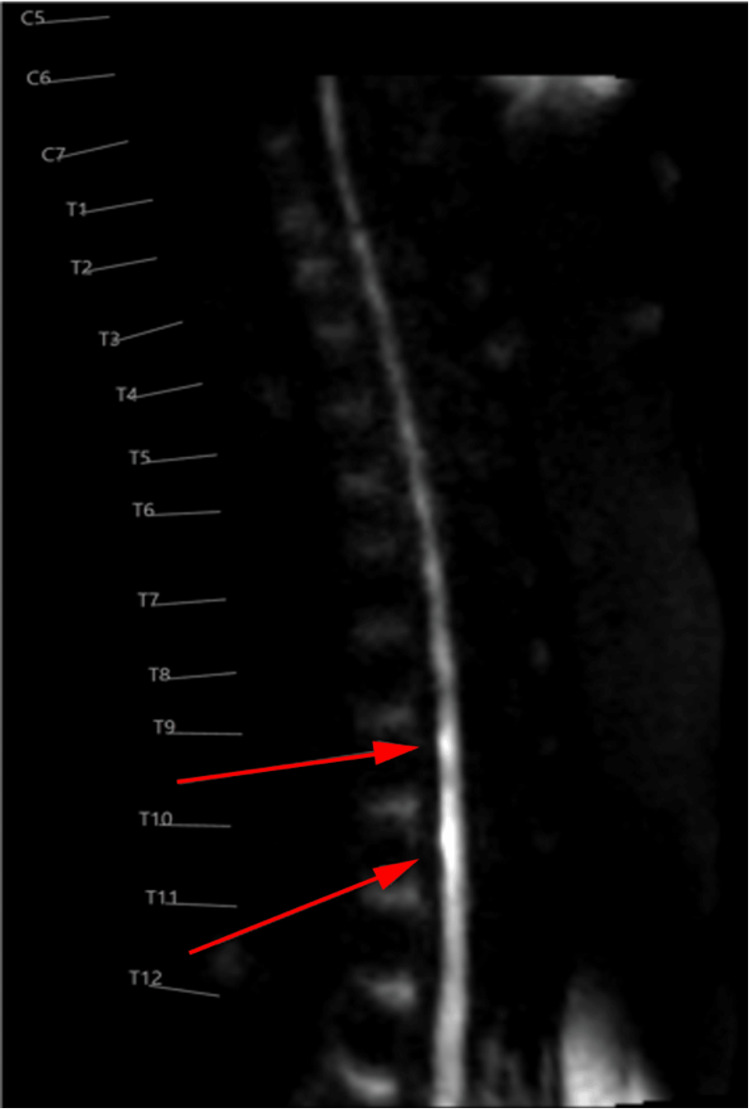
Sagittal DWI of the thoracic spine demonstrating diffusion restriction at the site of the signal abnormality from T8 to T9 through T11 to T12, indicated by red arrows DWI, diffusion-weighted imaging

**Figure 5:**
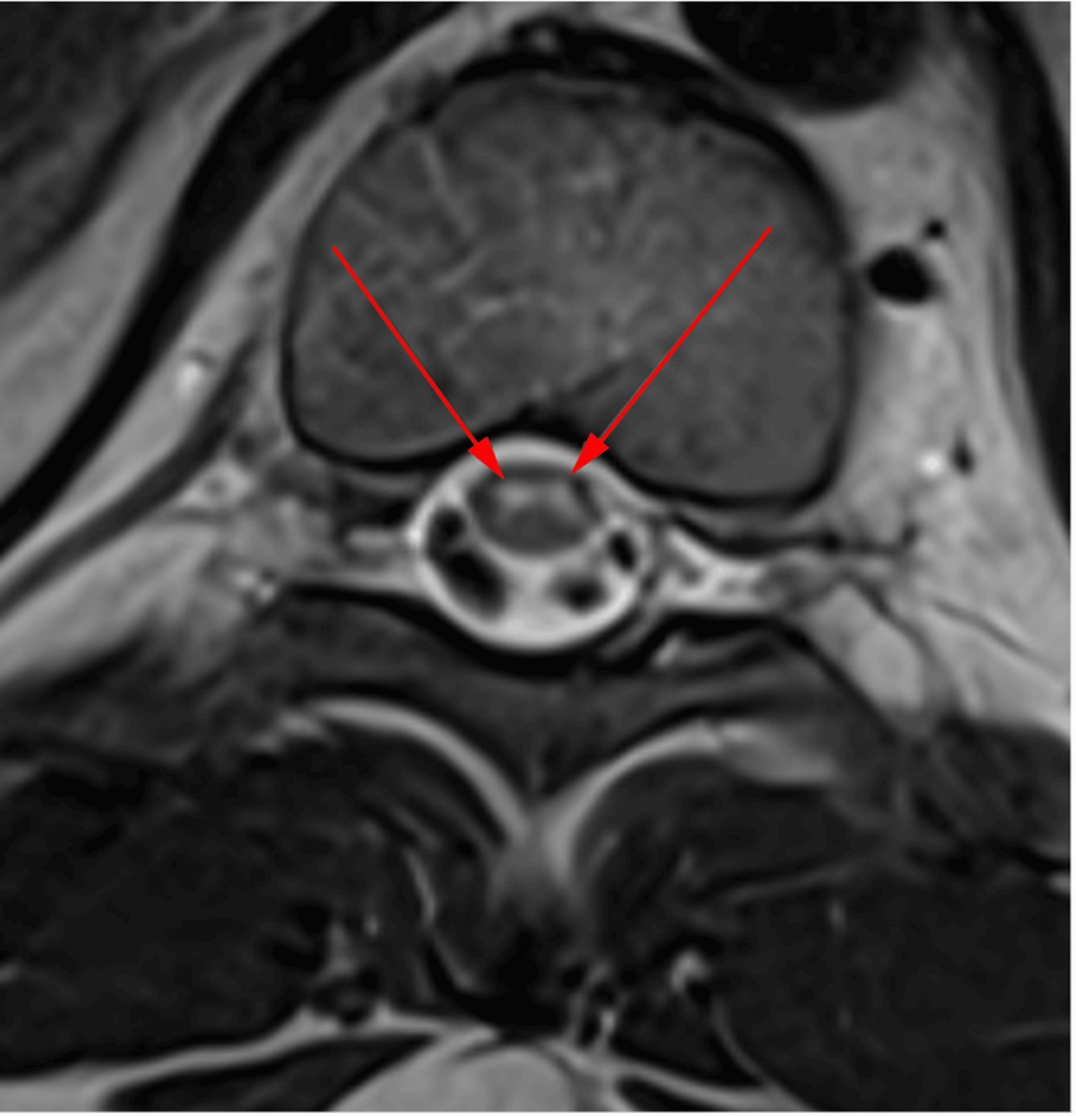
Axial T2-weighted MRI of the thoracic spine The red arrows demonstrate a hyperintense signal abnormality in the ventral spinal cord, representative of an Owl’s eye configuration.

The thoracic cord signal abnormality and T9-T10 transverse process fracture prompted further imaging using thin axial slices via diffusion-weighted imaging (DWI). Symmetric, ventral-predominant T8-T12 cord signal abnormality in an “Owl’s eye” configuration with restricted diffusion, consistent with SCI, was observed (Figure 4, Figure 5). Differential diagnoses included ASA infarction versus neuromyelitis optica, the latter rarely producing an Owl’s eye sign.

Over the course of the week, the patient gradually improved in BLE motor strength and sensory function. She was fitted with bilateral pressure-relief ankle-foot orthoses, allowing ambulation using a walker and gait belt. After an eight-day hospital stay, she was discharged to inpatient rehabilitation for one week, followed by twice-weekly outpatient rehabilitation.

At follow-up with neurology at two and five months post-injury, she demonstrated progressive improvement, with a narrow-based, well-balanced gait. Weakness persisted but was significantly improved compared to hospitalization. She was advised to continue physical therapy and to return in four months, or sooner if neurological symptoms recurred.

## Discussion

The ASA supplies the anterior two-thirds of the spinal cord. Although the spinal cord has robust vascularity, the mid-thoracic region is most vulnerable to infarction due to a smaller vessel diameter and weaker vascular supply from the unpaired artery of Adamkiewicz, which arises around T9-T12 and anastomoses with the ASA to supply the conus medullaris [4]. Approximately 75% of thoracolumbar spinal cord infarction cases present with an ASA infarction [5].

The mechanism of SCI following trauma is poorly understood, though three primary theories dominate. First, a more flexible vertebral column and less elastic spinal cord increase susceptibility to hyperflexion injuries, causing vascular spasm and cord insult [6]. Second, tearing of small spinal or radicular artery intimal linings can lead to dissection or thrombosis secondary to hematoma compression [4,7]. Third, fibrocartilaginous embolization of the nucleus pulposus can cause both traumatic and degenerative SCIs [8].

Compared to adults, extensive collateral blood flow in children renders their spinal cords less vulnerable to SCI [3]. The extent of disability in adulthood following pediatric SCI is evaluated using the American Spinal Injury Association impairment scale, which ranges from A (complete functional loss) to E (normal function). Approximately 50% of adults who experienced pediatric SCI are tetraplegic [9].

The median age of pediatric SCI is 14 years [9]. Traumatic ASA occlusion in pediatric SCI typically presents with acute back pain, followed by a symptom-free latent period, with neurological signs arising within 48 hours [1,7]. Three pediatric SCI case reports demonstrated normal BLE movement and sensation up to 48 hours prior to complete paraplegia [10]. In our patient, back pain and neurological symptoms began three days after the traumatic event. The literature describes eight cases of delayed symptom onset following SCI, ranging from two hours to four days, in children aged 1-10 years [7].

The gold standard for SCI diagnosis is MRI of the spine with axial thin-slice DWI [2]. Acute SCI on DWI demonstrates diffusion restriction, although imaging is often challenging due to the small spinal canal and cord diameter, respiratory motion, and CSF pulsation [4]. Spinal angiography can be useful for identifying occlusion of spinal cord vessels, particularly when MRI cannot resolve fine vascular details [7]. In this case, angiography was deemed unnecessary due to a patent artery of Adamkiewicz and a T9-T10 signal abnormality on MRI, indicating ASA occlusion. While smaller arterial occlusions could theoretically have been missed, this would not have altered treatment.

Our patient’s MRI of the thoracic spine with DWI demonstrated a classic Owl’s eye sign from T8 to T12. The Owl’s eye sign refers to bilateral symmetric T2 hyperintensities in the anterior horns of the spinal cord on axial MRI, indicating gray matter involvement. Spinal cord infarction is the most characteristic cause, particularly in the ASA territory. Acute flaccid myelitis, which typically presents with asymmetric flaccid weakness and is often associated with viral infections (not observed in this patient), also affects gray matter. The presence of a thoracic transverse process fracture favored trauma-related infarction. Neuromyelitis optica spectrum disorder less commonly shows the Owl’s eye sign compared to spinal cord infarction and typically presents with longitudinally extensive transverse myelitis affecting both gray and white matter [11]. Transverse myelitis may show central cord involvement, longitudinally extensive lesions, and variable enhancement, with or without the Owl’s eye pattern [12].

A markedly elevated OP of 54 mmHg, in the absence of inflammatory markers, supports pediatric ASA SCI as the most likely diagnosis, a finding not previously reported. The spinal cord is contained within a rigid vertebral canal, so edema following SCI increases intraspinal pressure [13]. Intracranial pressure (ICP) and intraspinal pressure are directly connected and equilibrate via the CSF system, with lumbar CSF pressure accurately reflecting ICP when CSF pathways are patent [14]. This relationship is governed by the Monro-Kellie doctrine, which states that the intracranial and spinal compartments form a continuous, closed system, wherein volume changes in one component must be compensated by reciprocal changes in others [15,16].

Although laminectomy and CSF drainage were considered to relieve pressure, these interventions were not pursued due to ongoing motor improvement and neurosurgical assessment showing a patent artery of Adamkiewicz, no compressive pathology, and no spinal instability. Fehling’s guidelines for adults with acute SCI recommend early laminectomy for cord decompression; however, two pediatric exploratory laminectomy cases were ineffective [7]. Emerging preclinical evidence suggests aspirin may exert neuroprotective effects in SCI models by reducing neuronal apoptosis and inflammation via the Nrf2/HO-1 pathway, though this has not been validated in humans [17].

Given alternative differentials, IV MPS 1,000 mg was administered daily for five days. This therapy may confer short-term motor improvement as a neuroprotectant and reduce secondary injury, but long-term benefits beyond six months are unproven [18].

Our patient maintained activities of daily living, though residual motor deficits persisted despite intensive physical therapy.

## Conclusions

Our case report highlights a thoracic ASA infarction diagnosed via axial thin-slice DWI, accompanied by elevated OP, a thoracic transverse process fracture, and lumbar paraspinal muscle strain, all managed conservatively with physical therapy. Although SCI is uncommon, its complications can be long-lasting and debilitating. A high index of suspicion for vascular insult to the spinal cord is warranted in any trauma, particularly involving the trunk, chest, or abdomen, to enable timely diagnosis and intervention. While spinal angiography was not performed in this patient, it may be useful in select cases to identify vasospasm, thrombus, vessel avulsion, or underlying vascular malformations. Further studies are needed to clarify optimal strategies for managing SCI.
